# Nanofocusing of structured light for quadrupolar light-matter interactions

**DOI:** 10.1038/s41598-018-26175-0

**Published:** 2018-05-17

**Authors:** Kyosuke Sakai, Takeaki Yamamoto, Keiji Sasaki

**Affiliations:** Research Institute for Electronic Science, Hokkaido University, Sapporo, Hokkaido, 001-0020 Japan

## Abstract

The spatial structure of an electromagnetic field can determine the characteristics of light-matter interactions. A strong gradient of light in the near field can excite dipole-forbidden atomic transitions, e.g., electric quadrupole transitions, which are rarely observed under plane-wave far-field illumination. Structured light with a higher-order orbital angular momentum state may also modulate the selection rules in which an atom can absorb two quanta of angular momentum: one from the spin and another from the spatial structure of the beam. Here, we numerically demonstrate a strong focusing of structured light with a higher-order orbital angular momentum state in the near field. A quadrupole field was confined within a gap region of several tens of nanometres in a plasmonic tetramer structure. A plasmonic crystal surrounding the tetramer structure provides a robust antenna effect, where the incident structured light can be strongly coupled to the quadrupole field in the gap region with a larger alignment tolerance. The proposed system is expected to provide a platform for light-matter interactions with strong multipolar effects.

## Introduction

The interactions of light and matter are of fundamental interest, and with the advent of photonic technologies, these interactions have become a flourishing field, stimulating basic science and consequent applications. Photonic crystals enable various control over light based on band structure engineering^1–3^, plasmonics provide a strong concentration of light with a large field enhancement^4–6^, and structured lights add an extra degree of freedom in angular momentum^7–9^. Most prominently, fundamental processes of light-matter interactions, i.e., absorption or emission, can be strongly modified by these photonic technologies.

For a long time, the interactions of light and matter have been known to be dominated by dipole-active transitions. Because the size (a_0_) of the wave function of the bound electron in atoms or solids is much smaller than the wavelength (λ) of light, this point-like wave function subjected to light experiences a uniform electric field. Thus, higher multipolar effects are normally considered to be too small and negligible, leading to the dipole approximation. The rates of electric quadrupole transitions are typically smaller than those of electric dipole transitions by a factor of α = (a_0_k)^2^, where k is the wavenumber of light (=2π/λ)^10^. In the optical region, typically, k = 2π/500 nm^−1^ and a_0_ = 0.1 nm, and therefore, α ≈ 10^−6^.

However, the validity of the dipole approximation is questionable under the use of photonic technologies. For a photonic crystal, for example, one can engineer the electromagnetic mode to have a specific orbital symmetry, where electron transitions that are usually allowed can be made forbidden, or more interestingly, electron transitions that are usually forbidden can be made allowed^3^, namely, selection rule modification. Several works have reported that the rates of quadrupole or even higher-order multipole transitions can be enhanced by the strong gradient of the electromagnetic mode in the near field of a dielectric microsphere^11^ or sub-micrometre aperture^12^ or in an evanescent field^13^. Notably, many works have reported that plasmonic systems can significantly enhance the multipolar effects because their k are much larger than that of light in the free space^14–19^.

Another scenario for selection rule modification has been argued in the field of structured light since the pioneering work of optical orbital angular momentum (OAM)^7^. Some forms of structured light, in particular, Laguerre-Gaussian LG^*l*^_*p*_ beams carry OAM of ±*lħ* per photon. Conventionally, the selection rules are interpreted for light with linear or circular polarization that possesses an angular momentum of ±*ħ*, but the total angular momentum can be augmented by OAM. This augmentation becomes particularly relevant for electric quadrupole transitions that require a change of two units of angular momentum (±2*ħ*) in the atom^20^. Several theoretical works have been reported discussing the quadrupole transition amplitude in the presence of OAM^21–23^. Recently, strongly modified selection rules for a quadrupole transition have been experimentally observed, showing that an atom can absorb two quanta of angular momentum from a single photon: one from spin (circular polarization) and another from OAM^24^.

It is now becoming clear that selection rules can be indeed modified, and multipolar effects should be of much more importance in light-matter interactions. However, to make the rate of multipolar transitions comparable to that of dipolar transitions, the coupling between the bound electron and the photon should be enhanced by spatially matching their wave functions. Here, we report a scheme to design the wave function of a photon (electromagnetic modes) to have a spatial distribution similar to the bound-electron wave function. In particular, we numerically demonstrate nanofocusing of structured light with a total angular momentum of ±2*ħ* using a plasmonic tetramer structure. We observe that the electromagnetic mode with a quadrupole profile is localized in and around the tetramer gap region of tens of nanometres, which is comparable to the size of relatively large bound-electron systems, e.g., quantum dots, excitons or cold atoms. We also report a tolerant structure design that allows an efficient coupling of incident structured light into the localized gap mode using a plasmonic crystal^25^.

## Results

### Plasmonic multimer system and incident structured light

**Figure 1:**
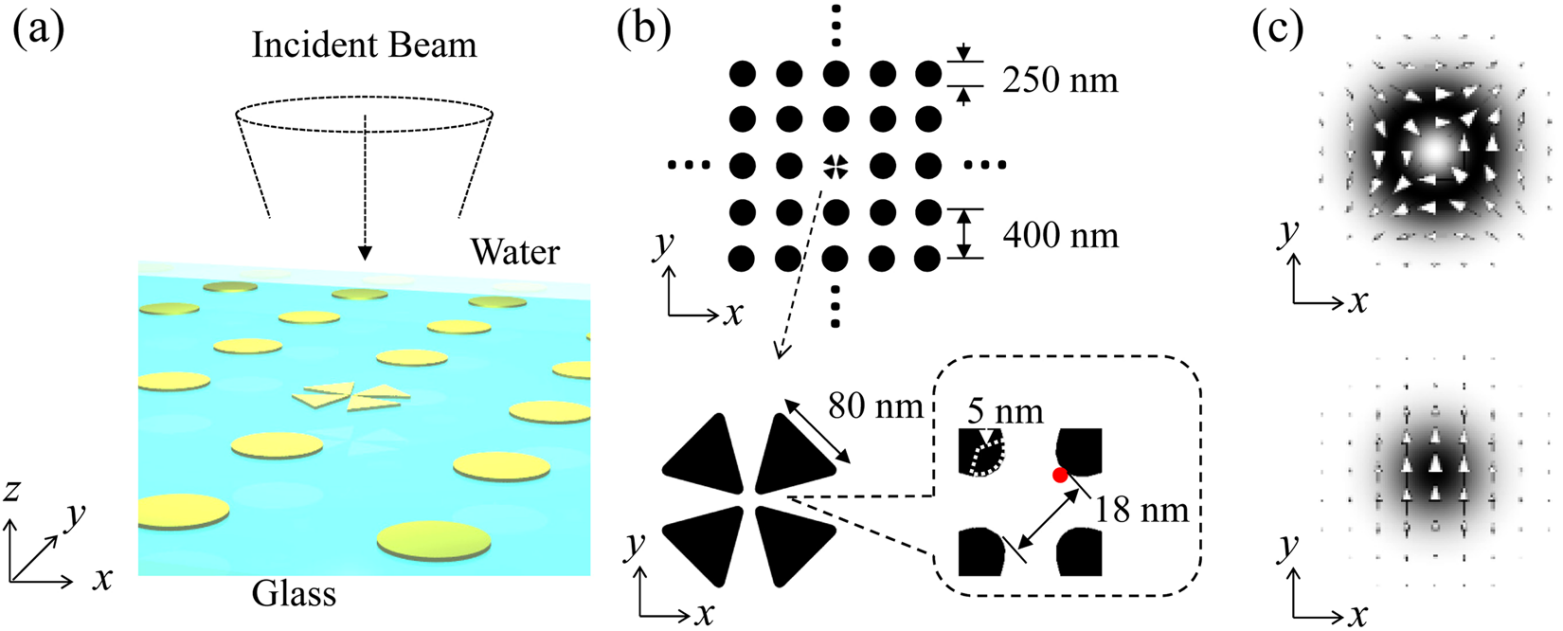
Calculation model of the plasmonic system. (**a**) The plasmonic system consisting of a gold tetramer structure surrounded by a gold nanodisk array is placed on a glass substrate in water. (**b**) Dimensions of the system. (**c**) Two types of incident light beams: a CV beam with a quadrupole profile and a linearly polarized Gaussian beam. The arrows indicate the electric field vectors, and the grey shading indicates the electric field norm distribution.

Our scheme is based on angular momentum transfer from photons to plasmons^26^. In particular, the total angular momentum of structured light can be transferred to a strongly localized plasmon mode in a multimer gap region^27^. Figure 1(a) shows the plasmonic system considered in this study. The tetramer structure consisting of four gold triangles and a gold nanodisk array (plasmonic crystal) are placed on a glass substrate in water, where an optical trapping technique should be applicable to transport the bound-electron systems, e.g., molecules or quantum dots, to the gap region. We set the refractive indices of glass and water as n_glass_ = 1.51, n_water_ = 1.33, respectively. We note that the present scheme is applicable to an air or vacuum environment instead of water. The complex refractive index of gold was taken from Johnson and Christy^28^. The gold tetramer structure is designed to ensure that plasmonic resonances occur in the near infrared region (~780 nm); the four equilateral triangles with side lengths of 80 nm are placed in a cloverleaf arrangement with 4-fold rotational symmetry to form a nanoscale gap at the centre, as shown in Fig. 1(b). The corners of the triangles are rounded with a curvature of 5 nm. The diagonal distance of the gap is 18 nm. The structural parameters of the plasmonic crystal are designed to match the lattice resonance with the plasmon resonance of the tetramer structure: a disk diameter of 250 nm, and a lattice period of 400 nm. The thickness of all elements is set to 30 nm. The near-field resonance spectrum is measured in the hot spot of the tetramer gap region situated 1 nm from the sidewall, as indicated by the red point. All visualizations including electric field distributions were performed on the plane at half the structure height. To transfer the angular momentum of the incident light to the plasmonic field excited in the tetramer structure, we used the focused structured light with an optical axis vertical to the glass surface. Among many types of structured light, we used a cylindrical vector (CV) beam that has a quadrupole profile, as shown in Fig. 1(c) (Methods). For comparison, we also used the linearly polarized Gaussian beam.

### Localized plasmon mode in the gap region of an isolated tetramer

**Figure 2:**
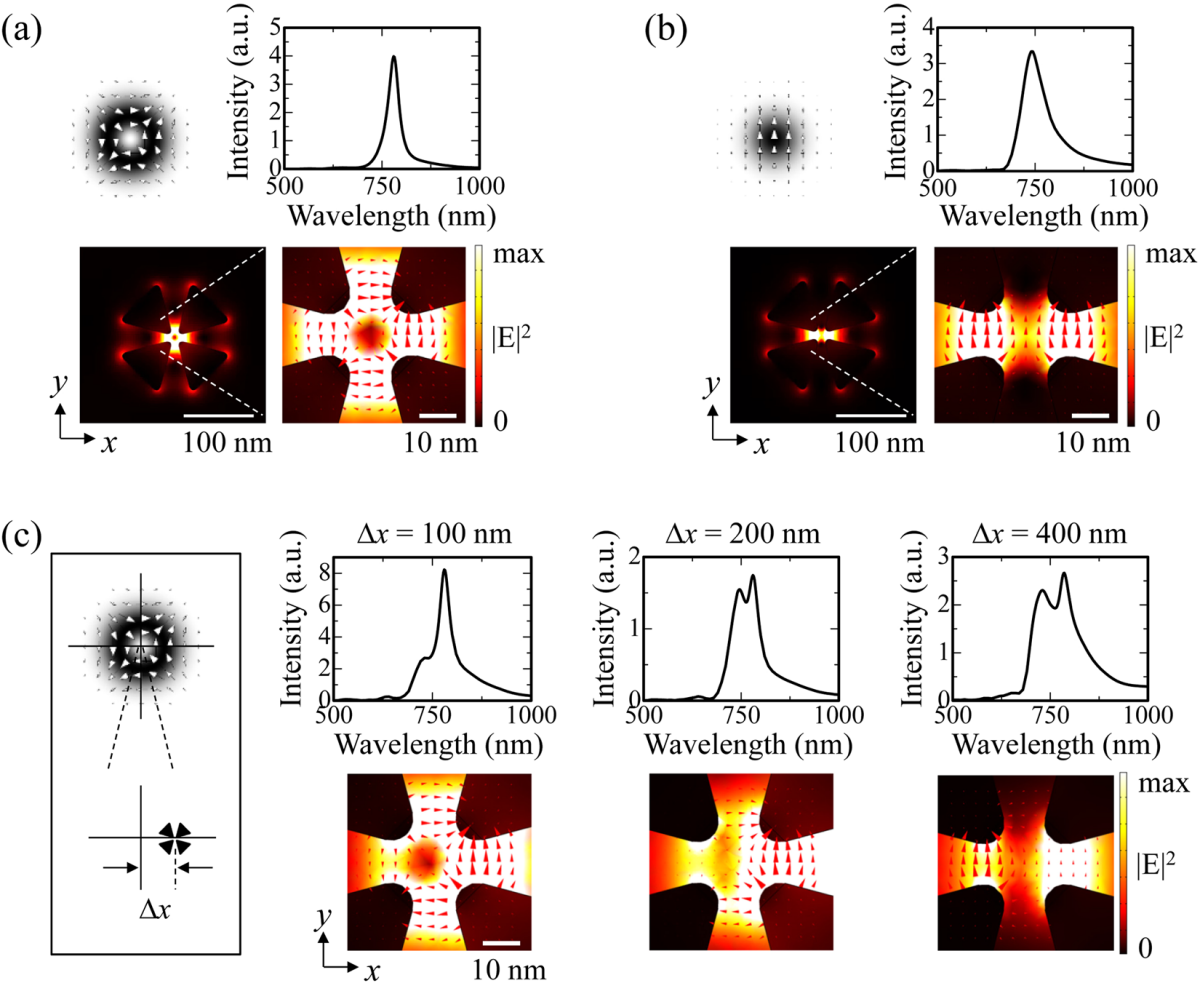
Plasmonic resonances in the isolated tetramer structure. (**a**) The near-field resonance spectrum and the near-field intensity distribution at the peak wavelength of 780 nm with the incident light of the CV beam. (**b**) The near-field resonance spectrum and the near-field intensity distribution at the peak wavelength of 740 nm with the incident light of the Gaussian beam. (**c**) The transition of the near-field resonance spectrum and the near-field intensity distribution at 780 nm with respect to the relative position (∆*x*) between the centre of the tetramer and the CV beam axis depicted in the inset. The red triangles indicate the electric field vectors.

We first consider the case for an isolated tetramer structure without the surrounding nanodisk array. When the CV beam is incident on the tetramer structure, part of the light can couple to the plasmon mode possessing near-field resonance spectral peaks at 780 nm, as shown in Fig. 2(a). The near-field intensity distribution at the peak wavelength also depicted in Fig. 2(a) clearly shows that the quadrupole field is squeezed into a nanoscale gap region ~20 nm. However, this result can only be obtained if the incident CV beam axis, i.e., singular point, overlaps the tetramer structure. Otherwise, the tetramer structure only experiences the nearly uniform electric field, similar to the case for the linearly polarized Gaussian beam. Under the Gaussian beam excitation, the resonant peak appears at a slightly shorter wavelength of 740 nm, and the near-field intensity distribution shows the dipole profile with linear polarization, as shown in Fig. 2(b).

Figure 2(c) shows the transitions of the plasmon resonances upon deviating the centre of the tetramer structure from the incident CV beam axis. As the deviation increases, the intensity of the shorter wavelength peak grows in the near-field spectrum. Accordingly, the near-field intensity distributions at 780 nm change to a dipolar profile. At a deviation of 400 nm, the electric field no longer retains a quadrupole profile.

### Efficient and robust system with a plasmonic crystal

**Figure 3:**
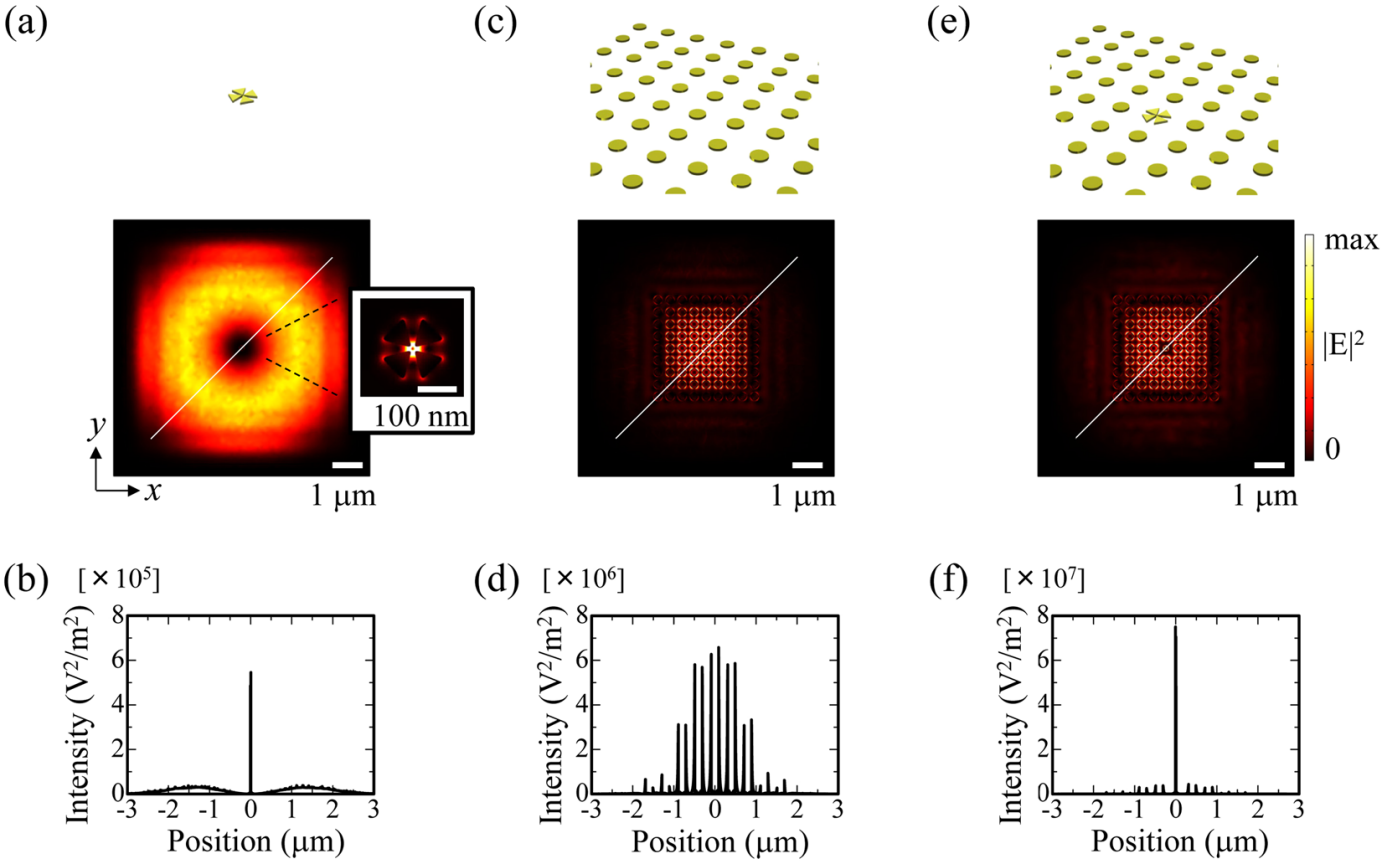
Combination of the tetramer structure and a plasmonic crystal. (**a**) The electric field intensity distribution for the isolated tetramer structure. The inset shows the near-field intensity distribution identical to the one in Fig. 2(a). (**b**) The intensity profile along the diagonal line of Fig. 3(a). The position origin is the centre of the structure. (**c**) The electric field intensity distribution of the plasmonic crystal. (**d**) The intensity profile along the diagonal line. (**e**) The electric field intensity distribution of the combined structure. (**f**) The intensity profile along the diagonal line. All the fields are taken at the plasmon resonance peak at 780 nm.

In practice, the alignment requires quite the effort to overlap the incident CV beam and the tetramer structure with a typical optical microscope. Efficiently coupling the incident light with the plasmonic field in the gap region is also difficult, as the tetramer structure must be placed at the dark centre of the CV beam. Figure 3(a) shows the intensity distribution in the xy plane for the tetramer structure. The ring shape profile represents the beam cross section of the incident CV beam. An identical incident beam was used for all the calculations throughout this paper. Figure 3(b) shows the intensity profile along the diagonal line in Fig. 3(a). Interestingly, although only part of the incident light couples to the plasmon resonance of the tetramer structure, the hot spot at the gap region shows a much larger peak intensity than the incident ring-shaped beam passing through the surrounding free space.

To receive more of the incident beam and to circumvent the deformation of the gap field under misalignment, we introduce a plasmonic crystal consisting of a square lattice nanodisk array, as shown in Fig. 3(c). The plasmonic crystal under a specific lattice resonance condition strongly captures incident light and forms a quadrupole field throughout the crystal^25^. The near-field distribution of the plasmonic crystal shows that the incident CV beam is well captured and confined to the centre of the crystal structure. Consequently, the hotspots along the diagonal line show a single lobed envelope function peaking at the centre of the entire structure, as shown in Fig. 3(d). The restricted memory size of our computer limits the array size to 9 × 9, and the outer part of the incident light is scattered away. For a larger array size, more of the incident light should be captured in the crystal.

Combining the tetramer structure with the plasmonic crystal, we achieve an efficient and tolerant system, where a large part of the incident light is captured and confined into the tetramer gap region while retaining the quadrupole near-field profile. Figure 3(e) shows the combined system where the central nanodisk of the crystal is replaced with the tetramer structure. The intensity distribution shows a similar profile as that of the plasmonic crystal except for the centre tetramer area. The diagonal intensity profile shows that the electromagnetic field is strongly confined in the gap region of the centre tetramer structure, and the intensity of the profile is two orders of magnitude larger than that for the isolated tetramer structure, as shown in Fig. 3(f).

**Figure 4:**
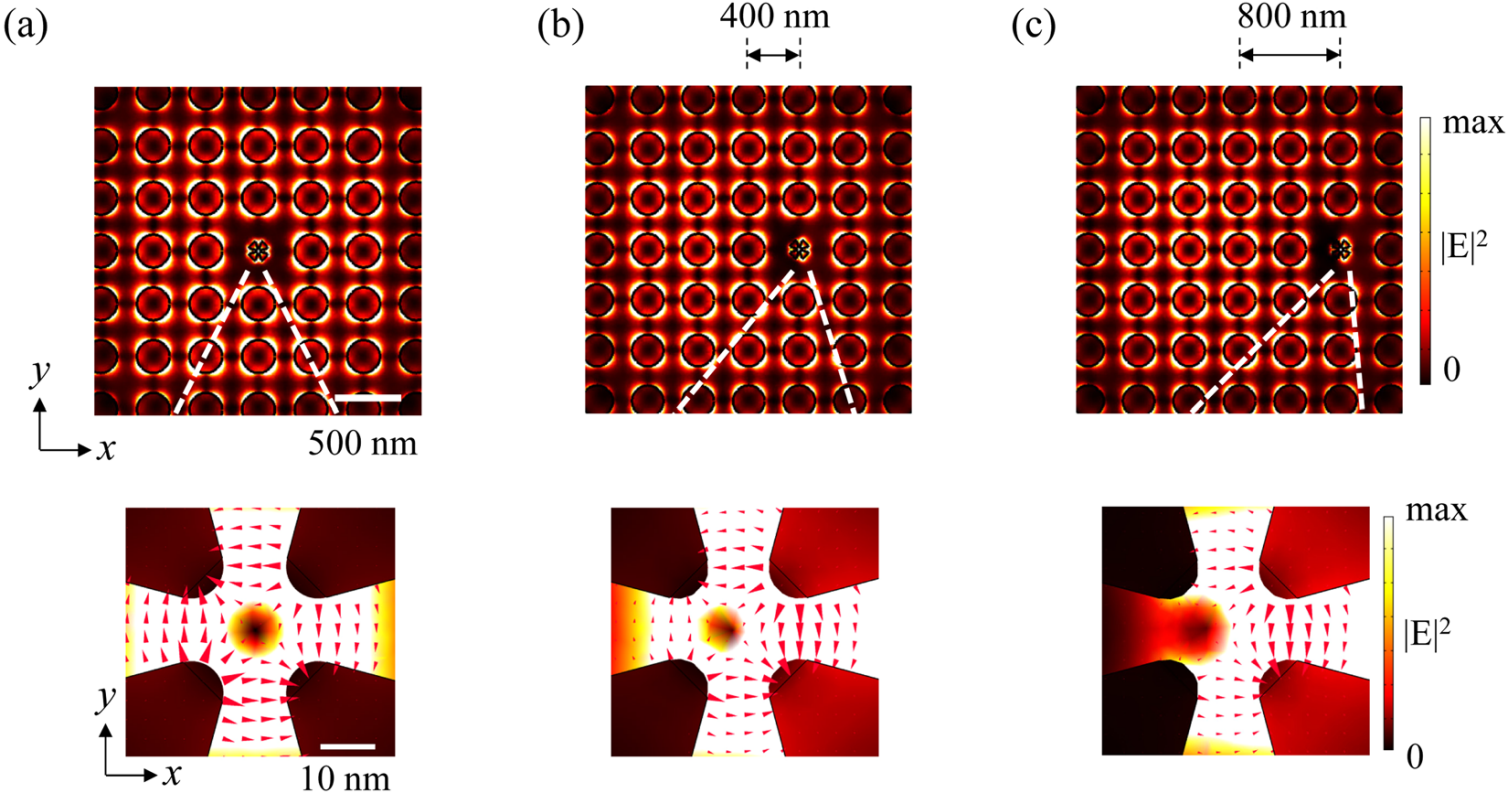
Position dependence of the tetramer structure in the plasmonic crystal. (**a**) The electric field intensity distribution with the tetramer structure placed at the centre of the plasmonic crystal. (**b**) The electric field intensity distribution of the tetramer structure shifted by one period in the x direction. (**c**) The electric field intensity distribution of the tetramer structure shifted by two periods in the x direction. All the fields are taken at the plasmon resonance peak of 780 nm.

The combined system also increases the alignment tolerance. Figure 4 shows the near-field distributions for the three cases, where the tetramer structure shifts the position from the centre of the crystal, i.e., centre of the incident beam, to the neighbouring nanodisks. With a shift of one period, i.e., 400 nm, the quadrupole field is retained in the tetramer gap region with a slight distortion, as shown in Fig. 4(b). Compared to the isolated tetramer structure, where the 400 nm deviation completely destroyed the quadrupole field, the combined system clearly possesses better tolerance against misalignment. At a deviation of 800 nm, the field distribution in the gap region shows a distorted quadrupole profile, as shown in Fig. 4(c), which is still far different from the dipole profile. With a shift to the diagonal direction, i.e., 400 nm or 800 nm in the x and y direction, the combined system also possesses better tolerance against misalignment (Supplementary Notes).

## Discussion

We numerically demonstrated nanofocusing of structured light with a quadrupole profile using a tetramer plasmonic structure. When the structured light is vertically incident on the tetramer structure, a quadrupole plasmonic field is excited and confined within a gap region of ~20 nm. However, the misalignment between the beam axis and the centre of the tetramer structure deteriorates the field profile of the quadrupole plasmonic resonance. A plasmonic crystal surrounding the tetramer structure circumvents this degradation and provides a robust antenna effect, where the incident structured light can be strongly coupled to the quadrupole field in the gap region. Qualitative consideration on the enhancement of the quadrupole transition^14,15,29^ and comparison with the dipole transition in the present system is a prerequisite for the future studies and applications. The proposed system should allow the plasmon wave function to have a spatial distribution similar to that of the bound-electron wave function, providing a platform for light-matter interactions with strong multipolar effects.

## Methods

### Calculation

We numerically calculated the electromagnetic field in our system using the finite-element method in the commercial software COMSOL Multiphysics with an RF module. We performed harmonic propagation calculations with perfectly matched layers (PMLs) on the side and lower boundaries with a scattering boundary condition on the top surface^26^ for both of the isolated tetramer and the combined systems. At the top surface, we excited the incident beam and made the beam propagate along the –z direction, normal to the glass surface. The incident beam was defined by the three components of the complex electric field (E_x_, E_y_, E_z_), given by vector diffraction theory^30,31^ (Supplementary Notes). To mesh our model, we used tetrahedral elements with varying dimensions. The maximum mesh size of the rounded corner sidewalls at the tetramer gap region was set to 3 nm, while that of the water and glass space was set to 150 nm.

### Incident beams

The CV beams were described by the sum of circularly polarized Laguerre-Gaussian LG^*l*^_*p*_(σ) beams, where σ is the handedness of the circular polarization (σ = ±1), giving rise to a spin angular momentum of σ*ħ* per photon. The CV beam with a quadrupole profile is given by LG^*l*^_*p*_(σ) beams with (*l* = 1, σ = 1) and (*l* = −1, σ = −1)^26^. The total angular momentum of each LG^*l*^_*p*_(σ) beam is 2*ℏ* and −2*ℏ*, and the coherent superposition of the two LG^*l*^_*p*_(σ) beams gives rise to a standing wave in the azimuthal direction that is linearly polarized, but the polarization direction is position dependent. Similarly, radially or azimuthally polarized beams, which are known to have interesting features at the focus^32^, can be described by the sum of LG^*l*^_*p*_(σ) beams with (*l* = 1, σ = −1) and (*l* = −1, σ = 1) with a respective phase difference between the two beams. Here, we confine our discussion to *p* = 0, that is, a single-ringed beam. The resultant expression for the CV beams is given in the Supplementary Notes.

### Data availability

All data generated or analysed during this study are included in this published article (and its Supplementary Notes).

## Electronic supplementary material

SUPPLEMENTARY NOTES

## References

[1] Yablonovitch E (1987). Inhibited spontaneous emission in solid-state physics and electronics. Phys. Rev. Lett..

[2] John S (1984). Electromagnetic absorption in a disordered medium near a photon mobility edge. Phys. Rev. Lett..

[3] Joannopoulos JD, Villeneuve PR, Fan S (1997). Photonic crystals: putting a new twist on light. Nature.

[4] Maier, S. A. *Plasmonics: Fundamentals and Applications* (Springer, 2007).

[5] Lal S, Link S, Halas NJ (2007). Nano-optics from sensing to waveguiding. Nat. Photon..

[6] Gramotnev DK, Bozhevolnyi SI (2013). Nanofocusing of electromagnetic radiation. Nat. Photon..

[7] Allen L, Beijersbergen MW, Spreeuw RJC, Woerdman JP (1992). Orbital angular momentum of light and the transformation of Laguerre-Gaussian laser modes. Phys. Rev. A.

[8] Allen, L., Barnett, S. M. & Padgett, M. J. *Optical Angular Momentum* (IOP, 2003).

[9] Rubinsztein-Dunlop H (2017). Roadmap on structured light. J. Opt..

[10] Blatt, J. M. & Weisskopf, V. F. *Theoretical Nuclear Physics* (John Wiley & Sons Inc., 1952).

[11] Klimov VV, Letokhov VS (1996). Quadrupole radiation of an atom in the vicinity of a dielectric microsphere. Phys. Rev. A.

[12] Von der Heydt A, Knorr A, Hanewinkel B, Koch SW (2000). Optical near-field excitation at the semiconductor band edge: Field distributions, anisotropic transitions and quadrupole enhancement. The Journal of Chemical Physics.

[13] Tojo S, Hasuo M, Fujimoto T (2004). Absorption enhancement of an electric quadrupole transition of cesium atoms in an evanescent field. Phys. Rev. Lett..

[14] Zurita-Sánchez JR, Novotny L (2002). Multipolar interband absorption in a semiconductor quantum dot I Electric quadrupole enhancement. J. Opt. Soc. Am. B.

[15] Kern AM, Martin OJF (2012). Strong enhancement of forbidden atomic transitions using plasmonic nanostructures. Phys. Rev. A.

[16] Jain PK, Ghosh D, Baer R, Rabani E, Alivisatos AP (2012). Near-field manipulation of spectroscopic selection rules on the nanoscale. Proc. Natl. Acad. Sci. USA.

[17] Takase M (2013). Selection-rule breakdown in plasmon-induced electronic excitation of an isolated single-walled carbon nanotube. Nat. Photon..

[18] Cotrufo M, Fiore A (2015). Spontaneous emission from dipole-forbidden transitions in semiconductor quantum dots. Phys. Rev. B.

[19] Rivera N, Kaminer I, Zhen B, Joannopoulos JD, Soljačić M (2016). Shrinking light to allow forbidden transitions on the atomic scale. Science.

[20] Franke-Arnold S (2017). Optical angular momentum and atoms. Philos. Trans. A Math. Phys. Eng. Sci..

[21] Schmiegelow CT, Schmidt-Kaler F (2012). Light with orbital angular momentum interacting with trapped ions. Eur. Phys. J. D.

[22] Lembessis VE, Babiker M (2013). Enhanced quadrupole effects for atoms in optical vortices. Phys. Rev. Lett..

[23] Afanasev A, Carlson CE, Mukherjee A (2016). High-multipole excitations of hydrogen-like atoms by twisted photons near a phase singularity. J. Opt..

[24] Schmiegelow CT (2016). Transfer of optical orbital angular momentum to a bound electron. Nat. Comm..

[25] Sakai K, Nomura K, Yamamoto T, Omura T, Sasaki K (2016). Quadrupole lattice resonances in plasmonic crystal excited by cylindrical vector beams. Sci. Rep..

[26] Sakai K, Nomura K, Yamamoto T, Sasaki K (2015). Excitation of multipole plasmons by optical vortex beams. Sci. Rep..

[27] Sasaki, K., Ide, M., Ishida, S. & Sakai, K. Plasmonic Nano-Shaping and Nano-Manipulation. *SPIE Nanoscience* + *Engineering*, San Diego, 10346–53 (2017).

[28] Johnson PB, Christy RW (1972). Optical constants of the noble metals. Phys. Rev. B.

[29] Filter R, Mühlig S, Eichelkraut T, Rockstuhl C, Lederer F (2012). Controlling the dynamics of quantum mechanical systems sustaining dipole-forbidden transitions via optical nanoantennas. Phys. Rev. B.

[30] Richards B, Wolf E (1959). Electromagnetic diffraction in optical systems. II. Structure of the image field in an aplanatic system. Proceedings of the Royal Society A: Mathematical, Physical and Engineering Sciences.

[31] Kitamura K, Sakai K, Takayama N, Nishimoto M, Noda S (2012). Focusing properties of vector vortex beams emitted by photonic-crystal lasers. Opt. Lett..

[32] Youngworth KS, Brown TG (2000). Focusing of high numerical aperture cylindrical-vector beams. Opt. Express.

