# Supplementary material for: Nanofocusing of structured light for quadrupolar light-matter interactions
Source: Sci Rep. 2018 May 17;8:7746. doi: 10.1038/s41598-018-26175-0 (PMC5958059; doi:10.1038/s41598-018-26175-0)
Supplement: Supplementary file 1 — SUPPLEMENTARY NOTES [file 41598_2018_26175_MOESM1_ESM.pdf]

SUPPLEMENTARY NOTES

Nanofocusing of structured light for quadrupolar light-matter interactions

Kyosuke Sakai*, Takeaki Yamamoto, Keiji Sasaki*

Research Institute for Electronic Science, Hokkaido University, Sapporo, Hokkaido, 001-0020 JAPAN, *E-mail: k_sakai@es.hokudai.ac.jp, sasaki@es.hokudai.ac.jp

Misalignment in the diagonal direction.

In addition to the effect of misalignment in the x direction, we considered the effect in the diagonal direction, i.e. 45 degree from the x axis. Figure S1 shows the transitions of the plasmon resonances. As the deviation increases, the intensity of the shorter wavelength peak grows in the near-field spectrum. Accordingly, the near-field intensity distributions at 780 nm change to a dipolar profile in a similar manner to the deviation in the x direction.

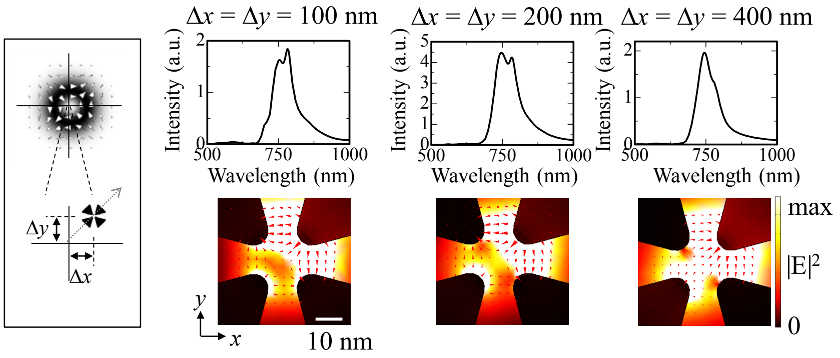

Fig. S1. The transition of the near-field resonance spectrum and the near-field intensity distribution at 780 nm with respect to the position shift (Δx and Δy) in the diagonal direction.

Figure S2 shows the near-field distributions for the three cases of the combined system, where the tetramer structure shifts the position in the diagonal direction. With a one-step shift, i.e., 400 nm in the x and y directions, the quadrupole field is retained in the tetramer gap region. Compared to the isolated tetramer structure, the combined system possesses better tolerance against misalignment also in the diagonal shift.

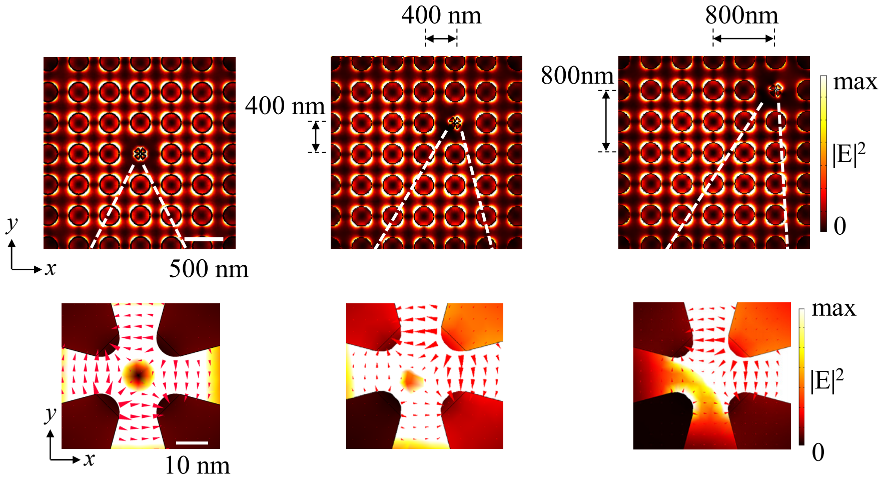

Fig. S2. Position dependence of the combined structure.

Incident beams.

The electric-field distribution in the cross-section of the CV beam or the Gaussian beam is expressed in the following form:

$$\mathbf{E}_{\text{beam cross.}} = u_{pl}(r,\phi)(e_r^{(0)}\mathbf{e}_r + e_\phi^{(0)}\mathbf{e}_\phi). \tag{s1}$$

The apodization function $u_{pl}(r, \phi)$ is given as[7]

$$u_{pl}(r,\phi) = \left(\frac{r\sqrt{2}}{w(0)}\right)^{|l|} L_p^{|l|}\left(2\frac{r^2}{w^2(0)}\right)\exp\left(-\frac{r^2}{w^2(0)}\right). \tag{s2}$$

Here, $w(0)$ is a radius of the beam waist, and $L^{|l|}_p$ is a Laguerre polynomial of the following form,

$$L_p^{|l|}(x) = \sum_{m=0}^{p}(-1)^m \frac{(p+l)!}{(p-m)!(l+m)!m!}x^m, \tag{s3}$$

where m is an integer, p and l are the mode indices for the radial and azimuthal directions, respectively. In Eq. (s1), $\mathbf{e}_r$ and $\mathbf{e}_\phi$ are the unit vectors of the electric field in the radial and azimuthal directions, respectively, $e_r^{(0)}$ and $e_\phi^{(0)}$ are their amplitudes. Here, we consider a specific type of CV beam that is linearly polarized at each local position, such that $e_r^{(0)}$ and $e_\phi^{(0)}$ are given as[31]

$$e_r^{(0)} = \cos\{(l-1)\phi+\psi\},\ e_\phi^{(0)} = -\sin\{(l-1)\phi+\psi\}, \tag{s4}$$

where ψ is the initial phase. The CV beam and the Gaussian beams shown in Fig. 1 (c) are given with $l=-1$, $\psi=\pi/2$ and $l=0$, $\psi=\pi/2$, respectively.

Using vector-diffraction theory[30, 32], the Cartesian components of the electric-field vector ($\mathbf{e}^{(s)}$) around the focus can be expressed in the following form:

$$\mathbf{e}^{(s)} = \begin{pmatrix} e_x^{(s)} \\ e_y^{(s)} \\ e_z^{(s)} \end{pmatrix} = \frac{-iA}{\pi}\int_0^{\alpha}\int_0^{2\pi} \sin\theta\cos^{\frac{1}{2}}\theta l_0(\theta) e^{ik(z_s\cos\theta+\rho_s\sin\theta\cos(\phi-\phi_s))}\mathbf{E}\,d\phi d\theta. \tag{s5}$$

Here, α is the maximum focusing angle (that is, the angle of a ray from the edge of the aperture), θ is the focusing angle, the cylindrical coordinates (ρ_s, ϕ_s, z_s) are defined at around the focus and $\mathbf{E}$ is the mode-dependent electric-field vector of the beam, given in the following form:

$$\mathbf{E} = \left\{ e_r^{(0)}\begin{pmatrix} -\cos\theta\cos\phi \\ -\cos\theta\sin\phi \\ \sin\theta \end{pmatrix} + e_\phi^{(0)}\begin{pmatrix} -\sin\phi \\ \cos\phi \\ 0 \end{pmatrix} \right\}. \tag{s6}$$

We note that the apodization function $l_0(\theta)$ is rewritten in the following form:

$$l_0(\theta) = \left(\frac{\sqrt{2}\beta_0 \sin\theta}{\sin\alpha}\right)^{|l|} L_p^{|l|}\left\{2\left(\frac{\beta_0\sin\theta}{\sin\alpha}\right)^2\right\}\exp\left\{-\left(\frac{\beta_0\sin\theta}{\sin\alpha}\right)^2\right\}. \qquad \text{(s7)}$$

Here, β_0 is the ratio of the pupil radius to the beam waist. In our calculation, the three components of the complex electric field (E_x, E_y, E_z) obtained in the above procedure are given to the top surface of our calculation model. The incident beam excited at the top surface propagates downward as shown in Fig. S3. The beam size is determined by α and β_0. We set α=11°and β_0=2 for both of the beams in the present study.

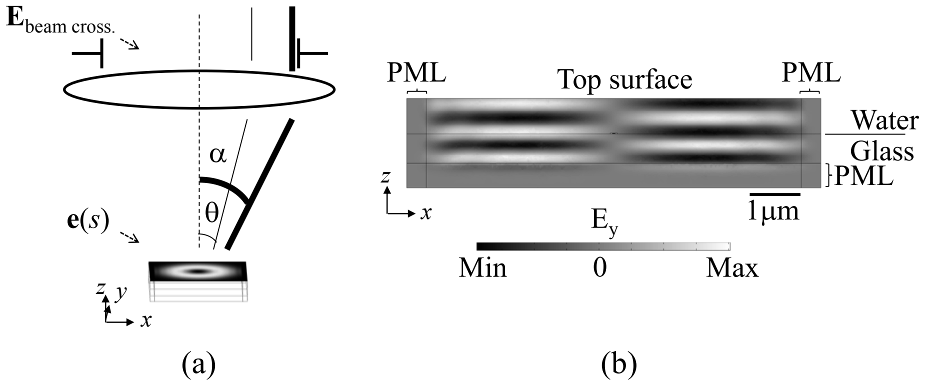

Fig. S3. (a) Schematic of the system in which the incident beams are determined. (b) The E_y in the x-z cross section of our calculation model. The CV beam is used as an incident beam.

The intensity distributions of the beam cross section on the plane at half the structure height are shown in Fig. S4. We note the tetramer structure was not included in these calculations. The full width at half maximum indicated by white dashed line is 4.4 μm for the CV beam and 3.2 μm for the Gaussian beam.

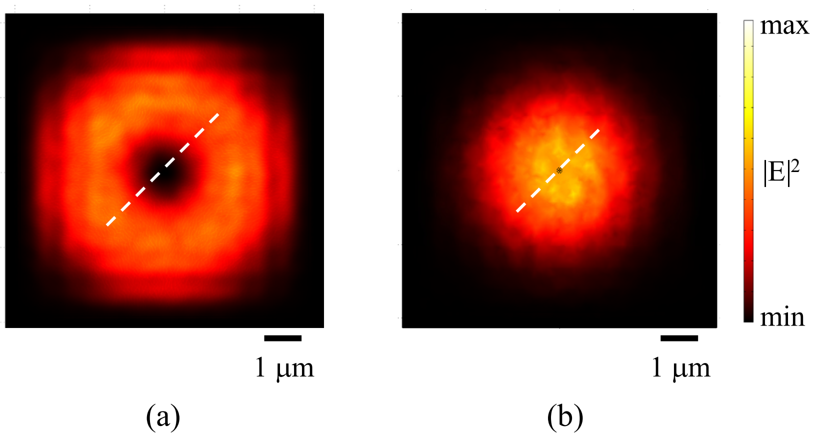

Fig. S4. The intensity distributions of the beam cross section for (a) the CV beam and (b) the Gaussian beam. The black line at the center of the Gaussian beam indicates the position of the tetramer structure.
